# Brief Mindfulness Meditation Improves Emotion Processing

**DOI:** 10.3389/fnins.2019.01074

**Published:** 2019-10-10

**Authors:** Ran Wu, Lin-Lin Liu, Hong Zhu, Wen-Jun Su, Zhi-Yong Cao, Shi-Yang Zhong, Xing-Hua Liu, Chun-Lei Jiang

**Affiliations:** ^1^Laboratory of Stress Medicine, Faculty of Psychology and Mental Health, Second Military Medical University, Shanghai, China; ^2^Counseling and Psychological Services Center, East China Normal University, Shanghai, China; ^3^Beijing Key Laboratory of Behavior and Mental Health, School of Psychological and Cognitive Sciences, Peking University, Beijing, China

**Keywords:** brief mindfulness meditation, randomized controlled trial, anxiety, depression, stress, emotion intensity, emotional memory, emotional attention bias

## Abstract

Mindfulness-based interventions have previously been shown to have positive effects on psychological well-being. However, the time commitment, teacher shortage, and high cost of classic mindfulness interventions may have hindered efforts to spread the associated benefits to individuals in developing countries. Brief mindfulness meditation (BMM) has recently received attention as a way to disseminate the benefits of mindfulness-based interventions. Most existing BMM methods are adaptations of the classic approach. Few studies have investigated the mechanisms underlying the beneficial effects of BMM. We developed a 15-min BMM named JW2016, which is based on the core concepts of mindfulness, Anapanasati (breath meditation of Buddhist Vipassana), our practical experience, and the results of scientific reports on meditation. We investigated the effects of this BMM on mood and emotion processing in an effort to create an effective, convenient, safe, and standardized BMM method that could benefit individuals with limited time or money to devote to meditation. Forty-six healthy participants (aged 18–25 years) were randomly allocated to the BMM group (*n* = 23) or the emotional regulation education (ERE) control group (*n* = 23). Forty-two of the study participants cooperated fully in all measurements and interventions (one time daily for seven consecutive days). Mood was measured with the Centre for Epidemiological Studies–Depression scale (CES-D) and the State Anxiety Inventory (SAI). Emotion processing was evaluated by assessing performance on an emotion intensity task, an emotional memory task, and an emotional dot-probe task. After intervention, the BMM group, but not the ERE group, showed a significant decreases in emotional intensity in response to positive as well as negative emotional stimuli, response time for emotional memory, and duration of attention bias toward negative emotional stimuli. Negative effects on mood state were found in the ERE group but not in the BMM group. This study demonstrated that BMM may improve aspects of emotion processing such as emotion intensity, emotional memory, and emotional attention bias. JW2016 BMM may be an effective, convenient, safe and standardized way to help practitioners remain focused and peaceful without any negative effect on emotion.

## Introduction

Mindfulness meditation is a form of self-regulatory exercise for mind and body. The core concepts of mindfulness include paying attention to the present moment and attaining a state of consciousness in a non-judgmental/accepting manner (Bishop et al., 2004; Lutz et al., 2008). Mindfulness meditation has its roots in Vipassana (insight meditation, a Buddhist meditation technique), which purports to affect mental events by engaging a specific attentional set (Lutz et al., 2008). As a clinical intervention, mindfulness meditation has been demonstrated to produce beneficial effects on mental and physical states, especially in terms of emotional improvement and recovery from affect-related psychopathology (Kabatzinn et al., 1992; Pinniger et al., 2012; Hoge et al., 2014; Khusid and Vythilingam, 2016). Mindfulness meditation has been proven to promote well-being and emotional balance (Krygier et al., 2013; Goyal et al., 2014), to decrease stress reactivity (Pace et al., 2009; Goyal et al., 2014), and to reduce negative feelings associated with anxiety and depression (Lane et al., 2007; Hoge et al., 2014; Khusid and Vythilingam, 2016).

Instead of attempting to change emotional experiences, meditation practice trains the individual to notice and observe emotions simply as they are and to accept emotional reactions as they arise (Lutz et al., 2008). Previous studies have explored the emotional benefits of meditation from numerous perspectives. Several studies have demonstrated that meditation may help to modulate emotional responses to negative stimuli (Erisman and Roemer, 2011; Johns and Medicine, 2015). Evidence from the field of cognitive neuroscience suggests that long-term meditation practice decreases the reaction intensity of the autonomic nervous system (Vasquez-Rosati et al., 2017) and attenuates the neural responses to emotional stimuli (Sobolewski et al., 2011; Taylor et al., 2011). Meditation training may also increase cognitive flexibility (Wenksormaz, 2005; Zeidan et al., 2011) and produce positive effects on emotion-cognition interactions. One study showed that an 8-week mindfulness-based stress reduction (MBSR) course enhanced attentional orientation and improved the ability to regulate emotion (Jha et al., 2007). An 8-week mindfulness-based meditation course reduced the attentional bias for pain-related threats in patients with fibromyalgia (Vago and Nakamura, 2011). Few studies have been conducted to examine the effects of meditation on positive emotion, and studies published to date have yielded conflicting findings. The results of previous studies showed that, after meditation, positive affect in response to a positive stimulus may increase (Erisman and Roemer, 2011), decrease (Ren et al., 2012), or remain unchanged (Sobolewski et al., 2011; Taylor et al., 2011). The effects of meditation on emotion-cognition interactions remain unclear.

Although mindfulness meditation training has proven to have many positive effects, the beneficiary population is relatively small because of the associated time commitment, teacher shortage, and high cost. Recently, brief mindfulness meditation (BMM) training has attracted increasing attention. One meta-analysis reported that BMM was more effective than control programs in decreasing negative affectivity (Schumer et al., 2018). However, the minimum amount of meditation training sufficient to improve emotional reactivity remains unknown, as does the mechanism underlying the positive effects reported in the literature. Previous studies found that 5 days of meditation (20 min daily) improved coordination of the body and mind in practitioners (Tang et al., 2009). Four days of mindfulness meditation training (20 min daily) decreased negative feelings such as fatigue and anxiety and improved cognitive functions such as visuospatial processing, working memory, and executive functioning (Zeidan et al., 2010b). Three days of 20-min mindfulness meditation sessions (1 h total) was more effective than sham mindfulness or control treatment in decreasing negative mood, depression, fatigue, confusion, and heart rate (Zeidan et al., 2010c). Even a single 10-min mindfulness intervention (Erisman and Roemer, 2011) or a 15-min focused-breathing meditation (Arch and Craske, 2006) may immediately decrease the intensity and negativity of emotional responses to affectively valenced external stimuli. Individuals may therefore benefit from very small doses of meditation training. However, other studies have reported conflicting conclusions. For example, researchers found that 7 days of meditation (30 min daily) reduced anxiety-related symptoms in practitioners but did not affect depression symptoms (Chen et al., 2013). 3 days of meditation (25 min daily) reduced self-reported psychological stress reactivity but increased salivary cortisol reactivity, as assessed with the Trier Social Stress Test (Creswell et al., 2014). Additional studies will be necessary to verify the specific effects and mechanisms of BMM.

BMM is not restricted by time or place and has the advantages of convenience and low cost. These characteristics suggest that the technique could benefit individuals who do not have enough time, money, or motivation to pursue other types of meditation training. Existing BMM methods are typically adapted from a more classical approach (e.g., Zeidan et al., 2010a; Collins et al., 2017). BMM methods developed independently of classical meditation practice are not common. Additional research will be necessary to determine the optimal time and frequency for the practice of BMM. Few studies have investigated the specific mechanisms underlying the effects of BMM. In this study, we developed a 15-min BMM based on the core concepts of mindfulness and Anapanasati (breath meditation related to Buddhist Vipassana), as interpreted in the context of our practical experience and that reported in other scientific reports on meditation. We performed a randomized controlled trial to investigate the effects of this BMM on mood state (depression and anxiety) and emotion processing (emotion intensity, emotional memory, and emotional attention bias). We hypothesized that BMM may be an effective, convenient, safe, and standardized approach to meditation that may improve mood and emotional processing among practitioners.

## Materials and Methods

This was a randomized controlled trial that included two groups: a BMM group (treatment group) and an emotional regulation education (ERE) group (comparison group). Both programs lasted for 1 week. Outcome measures were recorded at precise time points before and after engagement in the program. The entire experiment lasted for 3 weeks and consisted of three sessions: pre-test, intervention, and post-test. The present study was approved by the Committee on Ethics of Biomedicine Research at Second Military Medical University and registered in the Chinese Clinical Trial Registry (ChiCTR1800016081).

### Participation and Recruitment

The participants were recruited through campus advertisements during April and May 2017. We selected healthy people as participants to assess the efficacy and safety of meditation because there have been reports of the unwanted effects of meditation on practitioners (Cebolla et al., 2017). The following inclusion criteria were used: (1) 18–25 years of age; (2) undergraduate or graduate student; (3) in good health, with no mental illness according to established diagnostic criteria (DSM-IV-TR and ICD-10 combined); (4) ability to understand Cantonese; (5) willing to attend the BMM or ERE program.

The exclusion criteria were: (1) suffering from serious physical or mental illness or conditions expected to severely limit participation or adherence (e.g., pregnancy); (2) major life event (e.g., bereavement) or significant fluctuation in mood within the past month; (3) screening positive for major depression when evaluated with a structured diagnostic interview; (4) history of or interest in participation in a meditation program; (5) failure to participate in all scheduled sessions.

After the interview with the principal investigator, 46 students were recruited and randomly assigned to a treatment group with a list of computer-generated random numbers. Forty-two individuals completed all scheduled sessions: one dropped out because of illness; the others dropped out because they had an exam or class meeting and could not attend training. Table 1 shows the baseline demographic characteristics, for each intervention group and for the overall study population. Informed consent was obtained from all study participants.

**TABLE 1:** Baseline demographic characteristics by intervention group and total sample.

**Variable**	**Total Sample**	**BMM**	**ERE**
	**(*n* = 43)**	**(*n* = 22)**	**(*n* = 20)**
Age, mean (SD)	21.64 (2.14)	21.64 (2.34)	21.65 (1.95)
Gender (Female), No. (%)	32 (76.2)	18 (81.8)	14 (70)
Education (undergraduate), No. (%)	22 (52.4)	11 (50)	11 (55)

### Measurements

Measurements included the participants’ demographic information (age, sex, and education status), mood state (depression, anxiety) and emotion processing (emotion intensity, emotional memory, and emotional attention bias). Mood state and emotion processing were assessed before and after the program. Emotion processing was assessed using before and after testing, using different visual information each time.

#### Mood State

##### Depression

Depression was assessed using the CES-D, which investigated how often the participants had experienced specific depressive symptoms during the last week. Radloff (1977) originally proposed that the 20 items were categorized into four symptom groups: depressed affect (DA), somatic complaints (SC), interpersonal problems (IP), and positive affect (PA). Items were rated on a scale ranging from 1 (rarely or none of the time) to 4 (most or all of the time) (Radloff, 1977). The CES-D has been validated to be fit for Chinese adolescents and young adults (Radloff, 1991; Yen et al., 2000; Li and Hicks, 2010). However, in previous studies, Chinese students tended to have higher CES-D scores than members of the general population (Li and Hicks, 2010).

##### State anxiety

State anxiety (feelings of anxiety at a given moment) were assessed with a 20-item subscale of the State-Trait Anxiety Inventory (STAI-Form Y; Spielberger, 1983). Each item evaluated by the SAI is scored on a scale ranging from 1 (absent) to 4 (intense). The Chinese version of this test has been validated as a good psychometric index (Yan et al., 2014).

#### Emotion Processing

##### Emotion intensity

**FIGURE 1:**
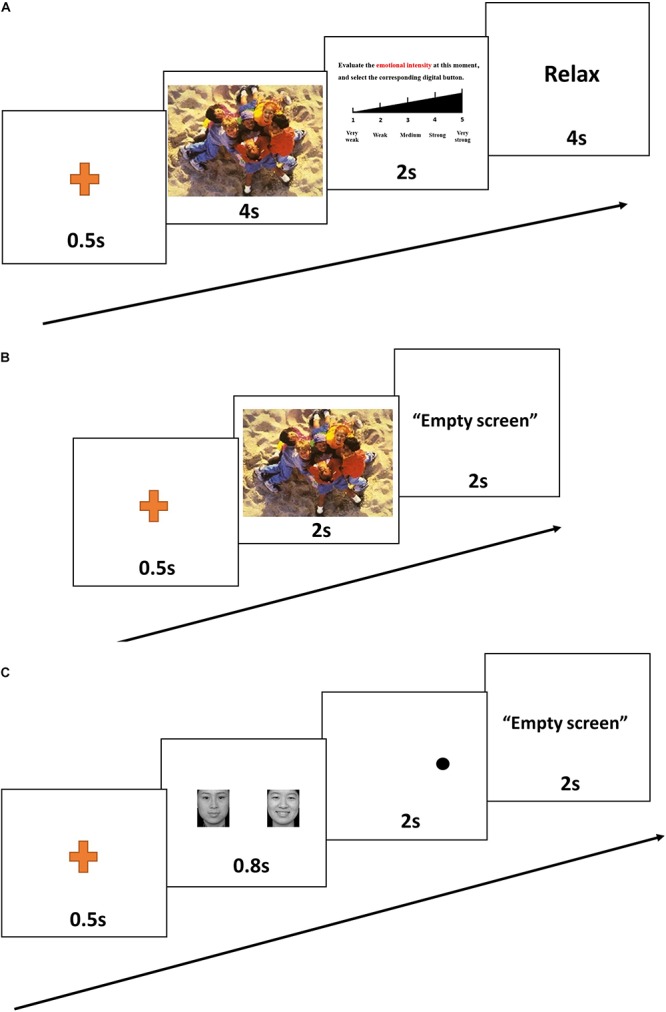
A representative trial from the emotion intensity task **(A)**, the emotional memory task **(B)**, and the emotional dot-probe task **(C)**.

An emotion intensity task was used to assess emotional intensity when participants were exposed to emotional stimuli (Ochsner and Gross, 2005; Taylor et al., 2011). The emotion-eliciting stimuli were selected from the International Affective Picture Set (IAPS; Center for the Study of Emotion and Attention [CSEA-NIMH], 1999). Pictures were selected to produce three distinct picture sets: positive, neutral, and negative. Each set included 66 pictures (6 for practice, 60 for evaluation). All pictures were randomly assigned to use in the pre- or post-test evaluation. For each of the three sets of pictures, there was no significant difference in pre- vs. post-test valence or arousal (Table 2). Each trial included four steps. First, a point for fixation appeared on the computer screen for 0.5 s. Second, a randomly selected image appeared on the computer screen. Participants were instructed to observe the image for 4 s and to try to remember it. Next, the image disappeared, and a Likert scale appeared. Participants were instructed to rate the strength of their emotional response on a scale of 1–5 (weak to strong). After the participants had rated their emotion intensity, the word “RELAX” appeared on the screen for 4 s, after which the next image appeared (Figure 1A).

**TABLE 2:** Valence and arousal of the pictures in the pre-test and post-test in the emotion intensity task [Mean (SD)].

		**Pre-test**	**Post-test**	***t*-test (*t*)**
Positive	Valence	7.04 (0.46)	7.06 (0.59)	–0.13
	Arousal	5.74 (0.36)	5.78 (0.67)	–0.25
Neutral	Valence	5.11 (0.44)	5.13 (0.70)	–0.10
	Arousal	4.57 (0.54)	4.59 (0.66)	–0.11
Negative	Valence	2.73 (0.64)	2.71 (0.73)	0.08
	Arousal	5.42 (0.76)	5.41 (0.65)	0.05

##### Emotional memory

After completing the emotion intensity task, participants were immediately asked to finish the emotional memory task (Groch et al., 2011). Recognition memory was assessed by asking participants to assign the term “familiar” or “novel” to each of 66 previously presented targets and 66 matched distractors (6 for practice, 60 for evaluation). The “novel” pictures were selected from the IAPS, which includes equal numbers of positive, neutral, and negative pictures. For all three sets of pictures, there was no significant difference in valence or arousal between pre- vs. post-test values, or between target vs. distractor pictures (Table 3). The picture remained on the screen until the participant had recorded his or her response (Figure 1B). Participants were asked to respond as correctly and as rapidly as possible. Accuracy and response time (correct responses only) were computed and analyzed.

**TABLE 3:** Valenceand arousal of the pictures in the pre-test and post-test in the emotional memory task [Mean (SD)].

		**Pre-test target**	**Post-test target**	**Pre-test distractor**	**Post-test distractor**	**ANOVA (pre-post), *F***	**ANOVA (tar-dis), *F***
Positive	Valence	7.04 (0.46)	7.02 (0.42)	6.98 (0.47)	7.06 (0.59)	0.00	0.16
	Arousal	5.74 (0.36)	7.06 (0.59)	7.02 (0.42)	6.98 (0.47)	0.03	0.22
Neutral	Valence	5.11 (0.44)	5.78 (0.67)	5.75 (0.54)	5.66 (0.60)	0.01	0.05
	Arousal	4.57 (0.54)	5.13 (0.70)	5.11 (0.44)	5.07 (0.61)	0.33	1.30
Negative	Valence	2.73 (0.64)	4.59 (0.66)	4.67 (0.57)	4.81 (0.75)	0.06	0.03
	Arousal	5.42 (0.76)	2.71 (0.73)	2.77 (0.61)	2.74 (0.68)	0.62	0.03

*Column “ANOVA (pre-post)” for main effect of pre/post test; Column “ANOVA (tar-dis)” for main effect of target/distractor pictures.*

##### Emotional attention bias

The attentional dot-probe task was used to assess emotional attention bias (Tsotsos et al., 1995). Three types of emotional faces taken from the Chinese Affective Picture System (CAPS) (Gong et al., 2011) were used for this task: 60 faces displaying negative emotion and 60 faces displaying positive emotion. Each face displaying a negative or positive emotion was paired with a matched neutral face. Another 60 neutral-neutral face pairs were used as filler. There were thus three types of face pairs: sad–neutral, happy–neutral, and neutral–neutral (60 pictures each). For each trial, a pair of faces (emotional–neutral) was presented on the screen, with one face on the left and the other face on the right. The emotional faces appeared in the right or left position with equal frequency. After fixation had been achieved, a given face pair was presented for 0.8 s, followed immediately by presentation of a probe in one of the two locations previously occupied by faces (Figure 1C). Participants were required to indicate the orientation of the dot by pressing a labeled key on the keyboard. The dot remained on the screen until the participant had recorded a response. For the “congruent” condition, the probe and the emotional face appeared in the same position. For the “incongruent” condition, the probe and the emotional face appeared in opposite positions. Attentional bias reaction time scores were calculated for each participant by subtracting the mean reaction time for congruent conditions from the mean reaction time for incongruent conditions (correct responses only).

### Intervention and Control

The interventions were single-blind in design; participants did not know the purpose of the experiment. A psychological counselor who has 10 years of mindfulness training and emotion education experience and knew the purpose of the experiment delivered the formalized program curriculum to participants in the two conditions. In order to reduce the potential bias, the leader conducted the intervention via audio instructions only, rather than providing one-on-one guidance.

#### Intervention: BMM

The BMM program used in this study was designed by a facilitator with more than 10 years of experience and training in meditation. This 15-min BMM (JW2016) was developed based on the core concepts of mindfulness and Anapanasati (Buddhist Vipassana breath meditation) (Chavan, 2007), in combination with knowledge gained from our practical experience and from scientific reports on meditation. We considered the validity and operability and tried to find a balance among practice time, frequency, and desired outcomes. Over 7 consecutive days, 22 participants attended training, in the same room and at the same time every day. On the first day of the meditation program, before meditation training, participants attended a 30-min lecture on mindfulness meditation theory. After that, participants sat on their cattail hassocks and followed audio instructions on how to practice the skills that would be tested. The audio instructions comprised 1 min of guided preparation and 15 min of mindfulness meditation training. Participants were instructed to close their eyes, relax, and focus on the flow of their breath. They were told to passively notice and acknowledge the thoughts arising randomly and to simply let “them” go, by bringing the attention back to the breath. As a manipulation check after each meditation session, each participant was asked “Did you feel that you were truly meditating?” Participants were allowed to ask questions about the meditation training. They were not asked to complete meditation homework or to practice outside of the intervention setting.

#### Control: ERE

The 20 participants in the ERE group received emotional education over the course of 1 week. On the first day, participants attended a 30-min lecture on emotion education and the emotion regulation theory proposed by Gross (1998). Participants were taught to recognize and regulate their own emotions. Participants then spent 15 min per day for 7 consecutive days promoting emotional awareness and regulation. No homework was given.

### Statistical Analysis

All statistical analyses were conducted with SPSS 21.0. Mood state and emotion processing scores were analyzed with an independent-sample *t*-test to test for between-group effects of intervention training and with a dependent-sample *t*-test to test for within-group effects (pre- vs. post-test). A significance level of 0.05 was used for all statistical tests.

## Results

### Mood State

The CES-D and SAI scores obtained are presented in Table 4. There was no significant effect of BMM training on mood state. Analysis of the post-test data revealed CES-D subscale and total scores that were significantly higher in the ERE group than in the BMM group. These differences reflected an increase in post-test CES-D scores in the ERE group (all *p* < 0.05) rather than a decrease in post-test CES-D scores in the BMM group (all *p* > 0.05). There was no significant difference in pre- vs. post-test scores within groups and no significant difference in SAI scores between groups. The results of a between-group *t*-test for mean changes between pre- vs. post-test scores showed that mean change in CES-D scores (four subscales and total scores) differed significantly between the ERE group and the BMM group. However, mean change in SAI was similar between groups.

**TABLE 4:** Results of pre-and post-test outcomes of mood state in two groups using *t*-test.

			**Mean (SD)**		**Mean change from pre-test (SD)**		***t*-test within group *t*, *d***		**Between-group *t*-test**	**Between-group *t*-test for mean change**
					
			**BMM**	**ERE**	**BMM**	**ERE**	**BMM**	**ERE**	***t*, *d***	***t*, *d***
CES-D	DA	pre	6.77 (5.34)	5.70 (3.99)					0.81, 0.25	
		post	5.77 (5.25)	13.40 (4.47)	−1.00(4.19)	7.76 (2.14)	1.12, 0.19	−15.80^∗∗∗^, −1.78	−5.16^∗∗∗^, −1.59	−8.58^∗∗∗^, −2.65
	PE	pre	7.32 (2.92)	7.95 (1.96)					−0.96, −0.30	
		post	8.05 (2.89)	10.10 (3.08)	0.73 (2.37)	2.24 (2.49)	−1.44, −0.25	−3.82^∗∗^, −0.79	−2.45^∗^, −0.76	−2.04^∗^, −0.63
	SC	pre	6.27 (3.60)	4.60 (2.04)					1.91, 0.59	
		post	5.41 (3.53)	10.15 (3.13)	−0.86(3.17)	5.62 (2.13)	1.28, 0.24	−11.47^∗∗∗^, −1.90	−4.74^∗∗∗^, −1.46	−7.84^∗∗∗^, −2.42
	IP	pre	1.36 (1.65)	0.75 (0.97)					1.57, 0.49	
		post	1.18 (1.37)	2.95 (1.32)	−0.18(1.44)	2.19 (0.93)	0.59, 0.12	−10.34^∗∗∗^, −1.81	−4.23^∗∗∗^, −1.31	−6.40^∗∗∗^, −1.98
	Total	pre	21.73 (8.10)	19.00 (4.90)					1.36, 0.42	
		post	20.41 (7.84)	36.60 (7.40)	−1.32(6.47)	17.81 (4.49)	0.96, 0.17	−17.49^∗∗∗^, −2.43	−7.09^∗∗∗^, −2.19	−11.21^∗∗∗^, −3.46
SAI	SAI	pre	46.05 (3.75)	47.20 (4.91)					−0.84, −0.26	
		post	47.82 (5.43)	44.55 (11.43)	1.77 (5.59)	−2.57(11.73)	−1.49, −0.38	0.99, 0.30	1.22, 0.38	1.56, 0.48

*^∗^0.01 < *p* < 0.05, ^∗∗^0.001 < *p* < 0.01, ^∗∗∗^*p* < 0.001.*

### Emotion Processing

The *t*-test was used to analyze the effects of BMM on emotion processing (Table 5). The results show that BMM training was effective in reducing emotional reaction intensity, in improving performance on tasks related to emotional memory, and in reducing attentional bias toward negative stimuli.

**TABLE 5:** Results of pre-and post-test outcomes of emotion processing in two groups using *t*-test.

			**Mean (SD)**		**Mean change from group pre-test (SD)**		***t*-test within group *t*, *d***		**Between- group *t*-test**	**Between-group for *t*-test mean change**
					
			**BMM**	**ERE**	**BMM**	**ERE**	**BMM**	**ERE**	***t*, *d***	***t*, *d***
Emotion intensity	Positive	pre	3.08 (0.80)	2.85 (0.72)					1.01, 0.31	
		post	2.75 (0.61)	2.76 (0.81)	−0.33(0.57)	−0.08(0.44)	2.70^∗^, 0.44	0.84, 0.11	−0.04, −0.01	−1.55, −0.48
	Neural	pre	2.32 (0.66)	2.17 (0.51)					0.91, 0.28	
		post	2.10 (0.50)	2.15 (0.48)	−0.22(0.64)	−0.01(0.35)	1.62, 0.37	0.17, 0.03	−0.35, −0.11	−1.30, −0.40
	Negative	pre	3.88 (0.45)	3.72 (0.57)					1.08, 0.33	
		post	3.67 (0.44)	4.02 (0.57)	−0.21(0.29)	0.29 (0.30)	3.36^∗∗^, 0.47	−4.39^∗∗∗^, −0.51	−2.21^∗^, −0.68	−5.50^∗∗∗^, −1.70
Emotional memory	Positive(R)	pre	0.93 (0.05)	0.86 (0.19)					1.73, 0.53	
		post	0.91 (0.05)	0.87 (0.20)	−0.02(0.05)	0 (0.26)	1.90, 0.49	−0.08, −0.02	−0.19, −0.06	−0.47, −0.15
	Positive(T)	pre	0.85 (0.08)	0.86 (0.11)					1.22, 0.38	
		post	0.78 (0.09)	0.83 (0.12)	−0.07(0.07)	−0.03(0.11)	4.58^∗∗∗^, 0.86	1.05, 0.22	−0.23, −0.07	−1.64, −0.51
	Neural(R)	pre	0.89 (0.08)	0.84 (0.18)					1.54, 0.48	
		post	0.89 (0.06)	0.83 (0.17)	0 (0.08)	−0.01(0.23)	−0.28, −0.07	0.17, 0.05	−1.13, −0.35	0.25, 0.08
	Neutral(T)	pre	0.86 (0.08)	0.86 (0.12)					1.04, 0.32	
		post	0.80 (0.10)	0.82 (0.10)	−0.05(0.07)	−0.04(0.12)	3.17^∗∗^, 0.57	1.53, 0.37	−1.62, −0.50	−0.31, −0.10
	Negative(R)	pre	0.93 (0.05)	0.86 (0.19)					1.64, 0.51	
		post	0.92 (0.04)	0.86 (0.20)	−0.01(0.04)	−0.01(0.26)	1.17, 0.22	0.09, 0.03	−0.54, −0.17	−0.08, −0.02
	Negative(T)	pre	0.88 (0.08)	0.91 (0.12)					1.38, 0.43	
		post	0.81 (0.09)	0.83 (0.09)	−0.07(0.08)	−0.08(0.10)	4.22^∗∗∗^, 0.82	3.33^∗∗^, 0.73	−0.97, −0.30	0.28, −0.09
Emotional attention bias	Positive	pre	−0.010(0.016)	0 (0.018)					−2.01, −0.62	
		post	−0.004(0.016)	−0.012(0.025)	0.006 (0.024)	−0.011(0.025)	−1.34, −0.39	2.07, 0.58	1.00, 0.31	2.41^∗^, 0.74
	Negative	pre	0.010 (0.016)	0.003 (0.025)					1.17, 0.36	
		post	−0.010(0.014)	0.003 (0.024)	−0.020(0.024)	−0.006(0.025)	3.93^∗∗∗^, 1.32	1.15, 0.27	−1.34, −0.41	−1.89, −0.58

*^∗^0.01 < *p* < 0.05, ^∗∗^0.001 < *p* < 0.01, ^∗∗∗^*p* < 0.001.*

#### Emotion Intensity

The study groups did not differ at baseline in terms of emotion intensity (all *p* > 0.05). Positive and negative emotion intensity recognized by participants decreased significantly after intervention in the BMM group (positive: *t* = 2.70, *p* = 0.013, *d* = 0.44; negative: *t* = 3.36, *p* = 0.003, *d* = 0.47). Negative emotion intensity recognized by participants increased significantly after intervention in the ERE group (*t* = −4.39, *p* < 0.001, *d* = −0.51). After intervention, negative emotion intensity recognized by participants was significantly higher in the ERE group than in the BMM group (*t* = −2.21, *p* = 0.033, *d* = −0.68). Significant group differences in mean pre- vs. post-test change were observed for negative emotion intensity (*t* = −5.50, *p* < 0.001, *d* = −1.70).

#### Emotional Memory

Groups did not differ at baseline in terms of emotional memory or accuracy or emotional memory response time (all *p* > 0.05). There was no significant difference between pre- vs. post-test values or between groups in terms of the accuracy of emotional memory. However, response time was significantly affected by participation in the BMM program. In the BMM group, emotional memory response time was significantly faster in post-test, compared with pre-test (positive: *t* = 4.58, *p* < 0.001, *d* = 0.86; neutral: *t* = 3.17, *p* = 0.005, *d* = 0.57; negative: *t* = 4.22, *p* < 0.001, *d* = 0.82). In the ERE group, post-test values of negative emotional memory response time were significantly decreased, compared with pre-test values (*t* = 3.33, *p* = 0.004, *d* = 0.73). The between-group *t*-test revealed no significant change.

#### Emotional Attention Bias

In the attentional dot-probe task, no between-group difference in attentional bias was found for either pre-test or post-test values (all *p* > 0.05). Significant treatment effects on attentional bias toward negative emotion were observed in the BMM group (*t* = 3.93, *p* < 0.001, *d* = 1.32). Before intervention, participants in the BMM group showed attentional bias, characterized by longer first-fixation duration when viewing negative facial expressions. However, in the post-test evaluation, participants in the BMM group did not show attentional bias toward negative emotion. The between-group *t*-test for mean change revealed significant differences between groups in attentional bias toward positive emotion (*t* = 2.41, *p* = 0.021, *d* = 0.74). After intervention, attentional bias toward positive emotion increased in the BMM group but decreased in the ERE group. Neither group showed attentional bias toward positive emotion.

## Discussion

This was a randomized controlled trial that aimed to compare BMM with ERE in terms of the effects on mood state and emotion processing. BMM significantly affected the intensity of positive and negative emotions, emotional memory, and negative emotional attention bias. Negative impact on mood state was found in the ERE group, but not the BMM group. The present study demonstrated that JW2016 BMM (15 min a day for 7 consecutive days) is effective in improving emotion processing.

Emotional intensity, emotional memory, and emotional attention bias improved in the participants in the BMM group. After intervention, emotional intensity toward negative stimuli decreased in the BMM group compared with the ERE group, which was consistent with the results of previous studies (Arch and Craske, 2006; Erisman and Roemer, 2011; Johns and Medicine, 2015). Emotional intensity toward positive stimuli also decreased after BMM, compared with ERE training; however, this trend was not significant. These results are consistent with those reported by Ren, who found that meditation training could create a peaceful state of mind, but inconsistent with those reported by most previous studies on the topic. Most previous studies on the topic have indicated that meditation practice has no effect on positive emotional responses (Sobolewski et al., 2011; Taylor et al., 2011). In the BMM group, emotional memory response time significantly decreased under all emotion conditions. These findings may indicate that memory improved after BMM. However, in the ERE group, only negative emotional memory response time decreased significantly. This results suggests that ERE training strengthened memories of negative information. In the attentional dot-probe task, a substantial change in negative emotional attention bias was observed in the BMM group. This finding is consistent with the results of previous studies (Garland and Howard, 2013) and may indicate that participants reduced attention to negative information after the intervention.

No significant change in symptoms related to depression and state anxiety was observed in the BMM group after intervention. There are several possible explanations for these findings. Firstly, BMM may have a limited capacity to improve mood. BMM does not utilize effective aspects of psychotherapy, such as the therapeutic alliance, which are known to contribute to the therapeutic effect (Leuchter et al., 2014). The therapeutic alliance could be incorporated into BMM practice. Secondly, the duration of intervention may not have been long enough. In previous studies, emotional symptoms were alleviated after meditation training 25–30 min per day over 3–5 days (Chen et al., 2013; Creswell et al., 2014). If the daily practice time were to be shortened, the number of days required for practice would increase considerably (Berghoff et al., 2017). For example, stress declined after 2 weeks’ participation in a 10-min daily meditation training session, whereas the increase in self-compassion in this group was significantly less than the increase observed in the group that practiced meditation for 20 min daily (Berghoff et al., 2017). Thirdly, the participants in this study were healthy students without obvious symptoms of depression or anxiety. Meditation training was more effective in improving mood state in patients with affective disorders such as major depression and anxiety (Goyal et al., 2014; Fan et al., 2015; Jain et al., 2015). Ceiling effects may partly explain the limited improvement in mood state among healthy people. More research is needed to verify the hypothesis presented above.

In addition, we found that BMM training had no negative effect on participants’ emotional processing or mood. BMM may not only contribute to a peaceful state of mind but also improve cognitive functions such as emotional memory and attention. However, in the ERE group, depression and emotional intensity in response to negative stimuli increased after intervention. These results imply that ERE training could exacerbate the negative emotional experiences of participants. Training may increase emotional awareness and reflection. It may have been difficult for participants to learn to adjust their emotions over such a short period of time without one-on-one coaching. Educators should therefore pay more attention to the mood status of participants during ERE training and provide more emotional guidance during the early stages of ERE.

The mechanism by which BMM improves emotional processing may be complicated. From the neuroendocrine perspective, one of the paths linking brief meditation to emotional improvement may be the hypothalamus–pituitary–adrenal (HPA) axis. Numerous studies have found that meditation [a 4-day mindfulness meditation (Turakitwanakan et al., 2013) or 48 hr of Integrated Amrita Meditation (Vandana et al., 2011)] may decrease the stress response and levels of cortisol (the end-product of the HPA axis). Emotional regulation may predict the symptomatic stress response and the recovery of salivary cortisol (Krkovic et al., 2018). In healthy individuals, the adaptive emotion regulation strategy was associated with greater cortisol recovery after exposure to a stressor (Lewis et al., 2017). This finding suggests that adaptive emotional regulation predicts improved HPA regulation. Our unpublished research also verified that BMM may lower salivary cortisol levels in college students with high suicide risk. In terms of the neuroendocrine mechanism, meditation may improve emotion processing by improving regulation of the HPA axis. In addition, Carlson et al. (2004) found that 8-week MBSR could increase plasma dehydroepian-drosterone sulfate (DHEAS), which is the major secretary product of the human adrenal and acts as a buffer against stress-related hormones. From a neurobiological perspective, the findings presented above suggest that meditation may influence the function and structure of the brain. For example, participation in an 8-week MBSR course may reduce the response of the right amygdala to emotional stimuli (Desbordes et al., 2012) and increase the density of regional gray matter in the brain (Holzel et al., 2011). Some neurochemical studies have shown that long-term meditation may regulate the neurotransmitters closely related to emotion processing, such as dopamine (Jung et al., 2010) and serotonin (Solberg et al., 2004). However, there is currently no evidence that briefer meditation sessions have the same effects. More studies are needed to verify whether brief meditation sessions achieve the same effect.

The present study has some limitations. Firstly, we failed to include a blank control manipulation, such as a waiting list group. Current evidence suggests that emotion processing is a relatively stable condition that is unlikely to be improved naturalistically, while mood state is more susceptible to interference (Etkin et al., 2011; Rock et al., 2016). Secondly, the interventions were single-blind in design; the same counselor delivered the curriculum to participants in each of the two conditions, and knew the purpose of the experiment. Though the leader conducted the intervention via audio instructions without one-on-one guidance, it may still be difficult to avoid unintentionally more enthusiastic or thoughtful intervention in the BMM group. Thirdly, the 1-week duration of the meditation program used for this study may not have been sufficient to achieve the maximum improvement possible. Future studies could prolong the training time and test the effects of meditation at different time points. Fourthly, small sample size was another drawback of this study. A *post hoc* power analysis was conducted with G^∗^Power 3.1. All statistical decisions were made using an alpha level of 0.05. Based on the sample size of this study, the power to demonstrate significant differences in post-intervention emotion processing ranged from 0.74 to 0.99 in both groups. Except for the powers of three indicators (positive emotion intensity after BMM, 0.74; negative emotion intensity after BMM, 0.87; neutral emotion memory, 0.89), the powers of other indicators were >0.95. Fifthly, there were too few males in the sample, which limited the power of the sex difference and made the results more applicable to females. Last but not the least, although this study discussed the mechanism of the effect of BMM through inference, physiological indicators such as hormones were not measured, which limited our exploration of the mechanism underlying the effects of intervention.

## Conclusion

This study demonstrated that JW2016 BMM (15 min a day for 7 consecutive days) was able to improve emotion processing including emotion intensity, emotional memory, and emotional attention bias, without any negative effect on the emotions of healthy practitioners. This BMM method may be applied to the emotional self-care of healthy people and/or the emotional rehabilitation of patients with affective disorders. It could be an effective, convenient, safe, and standardized way to improve emotion processing and to remain focused and peaceful. If JW2016 BMM could help healthy people to improve their emotion processing, it may also benefit a broader population. More empirical studies will be needed to verify the effects of BMM. We will also work on popularizing BMM to benefit more people.

## Data Availability Statement

The data used to support the findings of this study are available from the corresponding author upon request.

## Ethics Statement

The studies involving human participants were reviewed and approved by Committee on Ethics of Biomedicine Research, The Second Military Medical University. The patients/participants provided their written informed consent to participate in this study.

## Author Contributions

RW and C-LJ were responsible for the development of this particular study. RW wrote the first draft. RW and L-LL participated in the study design and performed the data analyses. C-LJ, RW, and X-HL designed the meditation method. RW, L-LL, and HZ were the principal researchers for the tests and interventions. W-JS, Z-YC, and S-YZ were responsible for conception of the project, questionnaire design, and manuscript review. All authors read and approved the final manuscript.

## Conflict of Interest

The authors declare that the research was conducted in the absence of any commercial or financial relationships that could be construed as a potential conflict of interest.

## Abbreviations

BMM: brief mindfulness meditation group
CAPS: Chinese Affective Picture System
CES-D: Centre for Epidemiological Studies–Depression Scale
DA: depressed affect
DHEAS, dehydroepiandrosterone sulfate. DSM: diagnostic and statistical manual of mental disorders
ERE: emotional regulation education group
HPA axis: hypothalamus–pituitary–adrenal axis
IAPS: International Affective Picture Set
ICD: International Classification of Diseases
IP: interpersonal problems
MBSR: mindfulness-based stress reduction
min: minute
PA: positive affect
SAI: State Anxiety Inventory
SC: somatic complaints
STAI-Form Y: State-Trait Anxiety Inventory.

## References

[B1] Arch J. J. Craske M. G. (2006). Mechanisms of mindfulness: emotion regulation following a focused breathing induction. *Behav. Res. Ther.* 44 1849–1858. 10.1016/j.brat.2005.12.007 16460668

[B2] Berghoff C. R. Wheeless L. E. Ritzert T. R. Wooley C. M. Forsyth J. P. (2017). Mindfulness meditation adherence in a college sample: comparison of a 10-min versus 20-min 2-week daily practice. *Mindfulness* 8 1513–1521. 10.1007/s12671-017-0717-y

[B3] Bishop S. R. Lau M. Shapiro S. Carlson L. Anderson N. D. Carmody J. (2004). Mindfulness: a proposed operational definition. *Clin. Psychol. Sci. Pract.* 11 230–241. 10.1093/clipsy/bph077

[B4] Carlson L. E. Speca M. Patel K. D. Goodey E. (2004). Mindfulness-based stress reduction in relation to quality of life, mood, symptoms of stress and levels of cortisol, dehydroepiandrosterone sulfate (DHEAS) and melatonin in breast and prostate cancer outpatients. *Psychoneuroendocrinology* 29 448–474. 10.1016/s0306-4530(03)00054-4 14749092

[B5] Cebolla A. Demarzo M. Martins P. Soler J. Garcia-Campayo J. (2017). Unwanted effects: is there a negative side of meditation? A multicentre survey. *PLoS One* 12: e0183137. 10.1371/journal.pone.0183137 28873417 PMC5584749

[B6] Center for the Study of Emotion and Attention [CSEA-NIMH] (1999). *The International Affective Picture System Digitized Photographs.* Gainesville, FL: Center for the Study of Emotion and Attention.

[B7] Chavan D. V. (2007). Vipassana: the Buddha’s tool to probe mind and body. *Prog. Brain Res.* 168 247–253. 10.1016/S0079-6123(07)68019-4 18166399

[B8] Chen Y. Yang X. Wang L. Zhang X. (2013). A randomized controlled trial of the effects of brief mindfulness meditation on anxiety symptoms and systolic blood pressure in Chinese nursing students. *Nurse Educ. Today* 33 1166–1172. 10.1016/j.nedt.2012.11.014 23260618

[B9] Collins K. R. L. Stebbing C. Stritzke W. G. K. Page A. C. (2017). A brief mindfulness intervention attenuates desire to escape following experimental induction of the interpersonal adversity implicated in suicide risk. *Mindfulness* 8 1096–1105. 10.1007/s12671-017-0686-1

[B10] Creswell J. D. Pacilio L. E. Lindsay E. K. Warren K. (2014). Brief mindfulness meditation training alters psychological and neuroendocrine responses to social evaluative stress. *Psychoneuroendocrinology* 44 1–12. 10.1016/j.psyneuen.2014.02.007 24767614

[B11] Desbordes G. Negi L. T. Thaddeus W. W. Pace B. Wallace A. Raison C. L. (2012). Effects of mindful-attention and compassion meditation training on amygdala response to emotional stimuli in an ordinary non meditative state. *Front. Hum. Neur.* 6:292. 10.3389/fnhum.2012.00292 23125828 PMC3485650

[B12] Erisman S. M. Roemer L. (2011). A preliminary investigation of the effects of experimentally- induced mindfulness on emotional responding to film clips. *Emotion* 10 72–82. 10.1037/a0017162.A 20141304 PMC2868364

[B13] Etkin A. Egner T. Kalisch A. R. (2011). Emotional processing in anterior cingulate and medial prefrontal cortex. *Trends Cogn. Sci.* 15 85–93. 10.1016/j.tics.2010.11.004 21167765 PMC3035157

[B14] Fan L. Wang X. Jiang T. (2015). The effect of body-mind relaxation meditation induction on major depressive disorder : a resting-state FMRI study. *J. Affect. Disord.* 183 75–82. 10.1016/j.jad.2015.04.030 26001666

[B15] Garland E. L. Howard M. O. (2013). Mindfulness-oriented recovery enhancement reduces pain attentional bias in chronic pain patients. *Psychother. Psychosom.* 82 311–318. 10.1159/000348868 23942276

[B16] Gong X. Huang Y. X. Wang Y. Luo Y. J. (2011). Revision of the Chinese facial affective picture system. *Chin. Ment. Health J.* 25 40–46.

[B17] Goyal M. Singh S. Sibinga E. Gould N. Rowland-Seymour A. Sharma R. (2014). Meditation programs for psychological stress and well-being : a systematic review and meta-analysis. *JAMA Intern. Med.* 174 357–368. 10.1001/jamainternmed.2013.13018 24395196 PMC4142584

[B18] Groch S. Wilhelm I. Diekelmann S. Sayk F. Gais S. Born J. (2011). Contribution of norepinephrine to emotional memory consolidation during sleep. *Psychoneuroendocrinology* 36 1342–1350. 10.1016/j.psyneuen.2011.03.006 21493010

[B19] Gross J. J. (1998). Gross, J. J. The emerging field of emotion regulation: an integrative review. *Rev. Gen. Psychol.* 2 271–299. 10.1037//1089-2680.2.3.271

[B20] Hoge E. A. Bui E. Marques L. Metcalf C. A. Morris L. K. Robinaugh D. J. (2014). Randomized controlled trial of mindfulness meditation for generalized anxiety disorder: effects on anxiety and stress reactivity. *J. Clin. Psychiatry* 74 786–792. 10.4088/JCP.12m08083 23541163 PMC3772979

[B21] Holzel B. K. Carmody J. Vangel M. Congleton C. Yerramsetti S. M. Gard T. (2011). Mindfulness practice leads to increases in regional brain gray matter density. *Psychiatry. Res.* 191 36–43. 10.1016/j.pscychresns.2010.08.006 21071182 PMC3004979

[B22] Jain F. A. Walsh R. N. Eisendrath S. J. Christensen S. Rael Cahn B. (2015). Critical analysis of the efficacy of meditation therapies for acute and subacute phase treatment of depressive disorders: a systematic review. *Psychosomatics* 56 140–152. 10.1016/j.psym.2014.10.007 25591492 PMC4383597

[B23] Jha A. P. Jason K. Baime M. J. (2007). Mindfulness training modifies subsystems of attention. *Cogn. Affect. Behav. Neurosci.* 7 109–119. 10.3758/cabn.7.2.109 17672382

[B24] Johns M. Medicine H. (2015). Intensive meditation training influences emotional responses to suffering. *Emotion* 15 775–793. 10.1037/emo0000080 25938614

[B25] Jung Y. H. Kang D. H. Jang J. H. Park H. Y. Byun M. S. Kwon S. J. (2010). The effects of mind body training on stress reduction, positive affect, and plasma catecholamines. *Neurosci. Lett.* 479 138–142. 10.1016/j.neulet.2010.05.048 20546836

[B26] Kabatzinn J. Massion A. O. Kristeller J. Peterson L. G. Fletcher K. E. Pbert L. (1992). Effectiveness of a meditation-based stress reduction program in the treatment of anxiety disorders. *Am. J. Psychiatry* 149 936–943. 10.1176/ajp.149.7.936 1609875

[B27] Khusid M. A. Vythilingam M. (2016). The emerging role of mindfulness meditation as effective self-management strategy, part 1: clinical implications for depression, post-traumatic stress disorder, and anxiety. *Mil. Med.* 181 961–968. 10.7205/MILMED-D-14-00677 27612338

[B28] Krkovic K. Clamor A. Lincoln T. M. (2018). Emotion regulation as a predictor of the endocrine, autonomic, affective, and symptomatic stress response and recovery. *Psychoneuroendocrinology* 94 112–120. 10.1016/j.psyneuen.2018.04.028 29775874

[B29] Krygier J. R. Heathers J. A. Shahrestani S. Abbott M. Gross J. J. Kemp A. H. (2013). Mindfulness meditation, well-being, and heart rate variability: a preliminary investigation into the impact of intensive Vipassana meditation. *Int. J. Psychophysiol.* 89 305–313. 10.1016/j.ijpsycho.2013.06.017 23797150

[B30] Lane J. D. Seskevich J. E. Pieper C. F. (2007). Brief meditation training can improve perceived stress and negative mood. *Altern. Ther.* 13 38–44. 10.1378/chest.10-1160 17283740

[B31] Leuchter A. F. Hunter A. M. Tartter M. Cook I. A. (2014). Role of pill-taking, expectation and therapeutic alliance in the placebo response in clinical trials for major depression. *Br. J. Psychiatry J. Ment. Sci.* 205 443–449. 10.1192/bjp.bp.113.140343 25213159 PMC4248233

[B32] Lewis E. J. Yoon K. L. Joormann J. (2017). Emotion regulation and biological stress responding: associations with worry, rumination, and reappraisal. *Cogn. Emot.* 11 1–12. 10.1080/02699931.2017.1310088 28397544

[B33] Li Z. Hicks M. H. (2010). The CES-D in Chinese American women: construct validity, diagnostic validity for major depression, and cultural response bias. *Psychiatry Res.* 175 227–232. 10.1016/j.psychres.2009.03.007 20006386

[B34] Lutz A. Slagter H. A. Dunne J. D. Davidson R. J. (2008). Attention regulation and monitoring in meditation. *Trends Cogn. Sci.* 12 163–169. 10.1016/j.tics.2008.01.005 18329323 PMC2693206

[B35] Ochsner K. N. Gross J. J. (2005). The cognitive control of emotion. *Trends Cogn. Sci.* 9 242–249. 10.1016/j.tics.2005.03.010 15866151

[B36] Pace T. W. W. Negi L. T. Adame D. D. Cole S. P. Sivilli T. I. Brown T. D. (2009). Effect of compassion meditation on neuroendocrine, innate immune and behavioral responses to psychosocial stress. *Psychoneuroendocrinology* 34 87–98. 10.1016/j.psyneuen.2008.08.011 18835662 PMC2695992

[B37] Pinniger R. Brown R. F. Thorsteinsson E. B. Mckinley P. (2012). Argentine tango dance compared to mindfulness meditation and a waiting-list control: a randomised trial for treating depression. *Complement. Ther. Med.* 20 377–384. 10.1016/j.ctim.2012.07.003 23131367

[B38] Radloff L. S. (1977). The CES-D scale: a self-report depression scale for research in the general population. *Appl. Psychol. Meas.* 1 385–401. 10.1177/014662167700100306 26918431

[B39] Radloff L. S. (1991). The use of the Center for Epidemiologic Studies Depression Scale in adolescents and young adults. *J. Youth Adolesc.* 20 149–166. 10.1007/BF01537606 24265004

[B40] Ren J. Huang L. Zhang Z. X. (2012). Meditation makes a peaceful state of mind: people’s positive and negative emotional response can be reduced by meditation training. *Acta Psychol. Sin.* 30 627–646.

[B41] Rock P. Goodwin G. Wulff K. McTavish S. Harmer C. (2016). Effects of short-term quetiapine treatment on emotional processing, sleep and circadian rhythms. *J. Psychopharmacol.* 30 273–282. 10.1177/0269881115626336 26869012

[B42] Schumer M. C. Lindsay E. K. David Creswell J. (2018). Brief mindfulness training for negative affectivity: a systematic review and meta-analysis. *J. Consult. Clin. Psychol.* 86 569–583. 10.1037/ccp0000324 29939051 PMC6441958

[B43] Sobolewski A. Holt E. Kublik E. Wróbel A. (2011). Impact of meditation on emotional processing-A visual ERP study. *Neurosci. Res.* 71 44–48. 10.1016/j.neures.2011.06.002 21689695

[B44] Solberg E. E. Holen A. Ekeberg Ø Østerud B. Halvorsen R. Sandvik L. (2004). The effects of long meditation on plasma melatonin and blood serotonin. *Med. Sci. Monit.* 10 96–101. 14976457

[B45] Spielberger C. D. (1983). *State-Trait Anxiety Inventory (Form Y).* Palo Alto, CA: Mind Garden, 19.

[B46] Tang Y. Ma Y. Fan Y. Feng H. Wang J. Feng S. (2009). Central and autonomic nervous system interaction is altered by short-term meditation. *Proc. Natl. Acad. Sci. U.S.A.* 106 8865–8870. 10.1073/pnas.0904031106 19451642 PMC2690030

[B47] Taylor V. A. Grant J. Daneault V. Scavone G. Breton E. Roffe-vidal S. (2011). NeuroImage Impact of mindfulness on the neural responses to emotional pictures in experienced and beginner meditators. *Neuroimage* 57 1524–1533. 10.1016/j.neuroimage.2011.06.001 21679770

[B48] Tsotsos J. K. Culhane S. M. Kei Wai W. Y. Lai Y. Davis N. Nuflo F. (1995). Modeling visual attention via selective tuning. *Artif. Intell.* 78 507–545. 10.1016/0004-3702(95)00025-9

[B49] Turakitwanakan W. Mekseepralard C. Busarakumtragul P. (2013). Effects of mindfulness meditation on serum cortisol of medical students. *J. Med. Assoc. Thai.* 96 (Suppl.), 90–95. 23724462

[B50] Vago D. R. Nakamura Y. (2011). Selective attentional bias towards pain-related threat in fibromyalgia: preliminary evidence for effects of mindfulness meditation training. *Cogn. Ther. Res.* 35 581–594. 10.1007/s10608-011-9391-x

[B51] Vandana B. Vaidyanathan K. Saraswathy L. A. Sundaram K. R. Kumar H. (2011). Impact of integrated amrita meditation technique on adrenaline and cortisol levels in healthy volunteers. *Evid. Based Complement. Alternat. Med.* 2011 1–6. 10.1155/2011/379645 21318156 PMC3034982

[B52] Vasquez-Rosati A. Brunetti E. P. Cordero C. Maldonado P. E. (2017). Pupillary response to negative emotional stimuli is differentially affected in meditation practitioners. *Front. Hum. Neurosci.* 11:209. 10.3389/fnhum.2017.00209 28515685 PMC5413546

[B53] Wenksormaz H. (2005). Meditation can reduce habitual responding. *Altern. Ther. Heal. Med.* 11 42–58. 15819448

[B54] Yan Y. Lin R. Tang X. He F. Cai W. Su Y. (2014). The relationship between worry tendency and sleep quality in Chinese adolescents and young adults: the mediating role of state-trait anxiety. *J. Health Psychol.* 19 778–788. 10.1177/1359105313479628 23520344

[B55] Yen S. Robins C. J. Lin N. (2000). A cross-cultural comparison of depressive symptom manifestation: China and the United States. *J. Consult. Clin. Psychol.* 68 993–999. 10.1037//0022-006X.68.6.993 11142551

[B56] Zeidan F. Gordon N. S. Merchant J. Goolkasian P. (2010a). The effects of brief mindfulness meditation training on experimentally induced pain. *J. Pain* 11 199–209. 10.1016/j.jpain.2009.07.015 19853530

[B57] Zeidan F. Johnson S. K. Diamond B. J. David Z. Goolkasian P. (2010b). Mindfulness meditation improves cognition: evidence of brief mental training. *Conscious. Cogn.* 19 597–605. 10.1016/j.concog.2010.03.014 20363650

[B58] Zeidan F. Johnson S. K. Gordon N. S. Goolkasian P. (2010c). Effects of brief and sham mindfulness meditation on mood and cardiovascular variables. *J. Altern. Complement. Med.* 16 867–873. 10.1089/acm.2009.0321 20666590

[B59] Zeidan F. Martucci K. T. Kraft R. A. Gordon N. S. Mchaffie J. G. Coghill R. C. (2011). Brain mechanisms supporting modulation of pain by mindfulness meditation. *J. Neurosci. Off. J. Soc. Neurosci.* 31 5540–5548. 10.1523/jneurosci.5791-10.2011 PMC3090218 21471390

